# Supplementary material for: Prevalence of childhood polyvictimization, mental health outcomes, and associated risk and protective factors in Ethiopia and Uganda refugee settings
Source: PLoS One. 2026 Mar 18;21(3):e0330551. doi: 10.1371/journal.pone.0330551 (PMC12998827; doi:10.1371/journal.pone.0330551)
Supplement: Attachment — Submitted filename: Report_MuthuriEtAl_PLOSOne.pdf [file pone.0330551.s002.pdf]

Referee Report for:

Title: Prevalence of childhood polyvictimization, mental health outcomes, and associated risk and protective factors in Ethiopia and Uganda refugee settings (Manuscript Number: PONE-D-25-42079)

Authors: Stella Muthuri, George Odwe, Francis Obare, Peter Kisaakye, Dagim Habteyesus, Gloria Seruwagi, Yohannes Dibaba Wado, Yadeta Dessie, Bonnie Wandera, Caroline W. Kabiru, and Chi-Chi Undie.

The authors utilize a novel and rare survey dataset derived from a representative sample of children and young adults residing in refugee settlements in Uganda and Ethiopia. They examine the risk factors associated with experiencing "childhood polyvictimization" and assess "childhood polyvictimization" itself as a risk factor for mental illness. Using multivariate logistic regression, the authors confirm the intuitive risk factors for childhood polyvictimization, as well as the notion that childhood polyvictimization is a risk factor for mental illness. However, the paper could benefit from a clearer motivation for the choice of such a narrow research question. Unfortunately, without a strong theoretical/*ex ante* motivation for the research question, there is a significant risk that it will be interpreted as an *ex post* realignment of the research question following data analysis. Below are some comments for your reference:

1 Major Comments

- The introduction begins with a clearly defined term "violence against children (VAC)"; however, it immediately transitions to "polyvictimization" in the second sentence. There is a missing connection regarding "victimization," which is not explicitly defined. While this may be a nitpicky point, I believe it is crucial to the framing of the paper, its contributions, connections to the literature, and welfare and policy concerns; it is important for comprehension and precision. It is only on the second page, in the middle of the last paragraph (line 108), that polyvictimization is finally explained. It would greatly enhance comprehension and motivation to move the discussion of the various forms of violence considered in this paper (physical, sexual, and emotional) to the first two paragraphs.

- Since the paper centers on the core concept of childhood polyvictimization, it needs to be clearly defined and contrasted with monovictimization. Why is it necessary to separate exposure to violence—either directed towards the child or witnessed by the child—into different forms? Why is a child who suffers two episodes of sexual violence considered less victimized than a child who experiences one episode of sexual violence and one episode of physical violence? The previous two questions must be supported by psychological theory and robust empirical evidence, at least in the context of high-income countries

(HIC), where data may be more abundant.

- The analysis pools children (ages 13 to 17) and adults (ages 18 to 24), which is problematic for the following reasons:

  1. Children are asked about their current exposure to violence, while adults are asked about their exposure during childhood. These questions are used to calculate the polyvictimization score (0-5), which represents the sum of various types of violence to which individuals have been exposed.
  2. Close to 50% were not exposed to any violence; yet, those with a score of 0 or 1 are coded as "non-polyvictims," while those with scores of 2, 3, 4, and 5 are coded as "polyvictims." It seems odd to pool non-victims with mono-victims.
  3. The risk factors, such as food insecurity, are presumably contemporaneous. Thus, for adults, polyvictimization serves as a risk factor for food insecurity, as it has occurred in the past. Similarly, for adults, polyvictimization is more likely to be a risk factor for poor quality relationships with parents rather than the reverse. How is school enrollment coded for adults? Is it contemporaneous, or does it refer to their childhood?
  4. The term "disabled"—a significant risk factor—remains undefined. Is it disability from birth? From forced displacement? Could adults become disabled after childhood? Could disability result from victimization?
  5. The paper includes "emotional violence" as a form of violence. It seems more appropriate to classify this as verbal or psychological abuse and to reserve the term violence for physical assaults, including both sexual and non-sexual acts. It also seems problematic to group witnessing any instance of violence, whether occurring in the household or outside of it, with the previous forms of violence.
  6. Attitudes towards intimate partner violence are deeply intertwined with exposure, making the study of this factor as a risk for victimization less informative.
  7. In the ethical considerations section, the authors state that some of the children in the sample were emancipated. Thus, parental consent was required only for interviews conducted with non-emancipated children. In the context of household violence, this raises concerns regarding sample selection bias.

2 Minor Comments

- In all the tables, when there are only two mutually exclusive and exhaustive categories for the variable being reported, it suffices to report the statistics for only one category, such as "Yes." Additionally, I am unclear about the value added by reporting the t-tests between males and females across the various variables.

- Since all the independent variables are binary, it might be more interpretable to run linear regressions rather than logistic regressions. Alternately, instead of or in addition to the odds ratios, the marginal effect at the mean or another easily interpretable estimate could be provided.

- Since there is not much contextual emphasis, the analysis can be presented more concisely by pooling samples from both countries and controlling for a country dummy variable.

- Given the confounding associated with adult status, I recommend including interaction terms between the adult dummy variable and each of the risk factors.

- Below are some suggestions to tighten the exposition:

  1. A more concise and engaging title.
  2. Parsimonious introduction of technical or technical-sounding terms. For example, prevalence, risk, and protective factors are all restatements of the common term "risk factors."

     onsistent use of active voice. Minimal use of passive voice.
  3. Repeating terms consistently. For example, with reference to violence, consistently use "type" or "form" to ensure clarity in referencing the same concept.

- The conclusion section is overly broad and non-specific. The paper does not claim any causal effects and should ideally refrain from making policy recommendations based on its conclusions.
