# Supplementary material for: Prevalence of childhood polyvictimization, mental health outcomes, and associated risk and protective factors in Ethiopia and Uganda refugee settings
Source: PLoS One. 2026 Mar 18;21(3):e0330551. doi: 10.1371/journal.pone.0330551 (PMC12998827; doi:10.1371/journal.pone.0330551)
Supplement: Attachment — Submitted filename: Referee_Report_PLOS ONE.docx [file pone.0330551.s001.docx]

**Prevalence of childhood polyvictimization, mental health outcomes, and associated risk and protective factors in Ethiopia and Uganda refugee settings**

**Comments**

The paper investigates the prevalence of childhood polyvictimisation and mental health outcomes, and associated risk/protective factors in Ethiopia and Uganda refugee settings. The paper uses datasets from the Ugandan and Ethiopian Humanitarian Violence against Children and Youth Surveys (HVACS). Using cross-tabulation with chi-square and estimation of a multivariate logistic regression model, the findings reveal that polyvictimisation was significantly associated with higher odds of mental health distress, self-harm, and suicidal ideation/attempts among females and males in both Uganda and Ethiopia refugee settings, respectively.

The paper is well-written and focuses on interesting issues that are prevalent in conflict-affected countries. My specific comments based on my review of the paper are provided below:

(i) The paper indicates a number of studies that focus on the prevalence of polyvictimisation in developing countries. The contributions of the paper to the existing literature could have strengthened to go beyond – undertaking another study in that focuses on humanitarian settings (Uganda and Ethiopia). The justifications provided for another study isn’t sufficient to warrant publishing the paper in a reputable journal like PLOS ONE.
(ii) In the discussion of the data sources, there are some repetitions in the discussions of the data between line 138-145, and 161-164.
(iii) In addition to using an additive index for polyvictimisation, the study could conside the use of Principal Component Analysis (PCA) or Multiple Correspondence Analysis (MCA) to generate an index for polyvictimisation.
(iv) In the description of mental health measure, the study generates a threshold for less severe mental health score if the mental health score is less than 13, and moderate to severe mental health stress for scores of 13 points or higher. How did the study arrive at this threshold? Please provide evidence to lend credence to the cut-off adopted.
(v) In the empirical analysis, in addition to using chi-square (X^2^), the paper could have used test of mean difference to obtain difference in outcomes between polyvictimisation, and non-polyvictimisation.
(vi) It would have been nice to present the econometric model used in the empirical analysis of the multivariate regression model. The econometric model should be presented such that the outcome (dependent variable), and the regressors are indicated.
(vii) The empirical analysis could have gone beyond multivariate logistic regression to include a further empirical analysis such as propensity score matching (PSM) method that use matching of observable characteristics to obtain robust results compared to multivariate logistic regression.
