# Supplementary material for: Prevalence of childhood polyvictimization, mental health outcomes, and associated risk and protective factors in Ethiopia and Uganda refugee settings
Source: PLoS One. 2026 Mar 18;21(3):e0330551. doi: 10.1371/journal.pone.0330551 (PMC12998827; doi:10.1371/journal.pone.0330551)
Supplement: Attachment — Submitted filename: Response to Reviewers - PONE-D-25-42079.docx [file pone.0330551.s003.docx]

Dear Editor(s),

I am pleased to submit our detailed responses to the reviewers’ and editor’s comments, along with the revised manuscript. We have carefully addressed each point and made the necessary changes to strengthen the paper and ensure it meets the standards of *PLOS ONE*.

It is our hope that the revisions reflect thoughtful consideration of the valuable feedback provided and that the manuscript now meets the expectations of the journal.

We greatly appreciate the time and effort invested by the reviewers and editor in improving the quality of this work.

Thank you for your continued guidance throughout this process.

Kind regards,

Stella Muthuri, Phd

(on behalf of all co-authors)

**********

**PONE-D-25-42079: Prevalence of childhood polyvictimization, mental health outcomes, and associated risk and protective factors in Ethiopia and Uganda refugee settings.**

**Academic Editor’s Comments:**

(1) Having reviewed both the manuscript and the referee reports, I largely concur with the points raised by referees 1 and 3 that the findings for the adult population are hard to interpret and should probably be excluded.  First, we do not know if these individuals were displaced as children.  Second (and more importantly), the various risk and protective factors are measured contemporaneously (in adulthood), not retrospectively (at the time of the abuse), and thus the findings cannot be interpreted as evidence that any particular characteristic had a protective effect vis-a-vis polyvictimization: the victimization could have occurred long before any particular characteristic was determined.  I suggest you drop these findings.  I also concur with multiple points raised by the referees noting that a more robust defense of the definition of polyvictimization is required; and I also find that the differences between males and females seem somewhat overemphasized in the text (or at least, are overemphasized without any clear conceptual framework for understanding these differences).  Please respond carefully to all of the comments provided by all three referees.

**Response:** We appreciate the reviewer’s observation and agree that careful interpretation is essential when presenting results, particularly for participants aged 18–24 years. The HVACS collected information that clarifies the displacement history of households where respondents aged 13–24 reside. Specifically, the median duration of stay for household heads serves as a reasonable proxy for how long young people in the sample have lived in the camp environment. In Uganda, for 18–24-year-olds, the median duration was 6 years for both males and females, while in Ethiopia it was 11 years for females and 12 years for males. These figures indicate that most respondents have spent a substantial portion of their childhood or adolescence in settlements or camps.

The survey also asked older respondents about the timing of their first incident of childhood violence, which is critical for understanding whether violence occurred before or after displacement. In Uganda, among 18–24-year-olds who experienced sexual violence before age 18, 73.3% of females and 53.2% of males reported that the first incident happened after arrival in the settlement. For physical violence, 66.6% of females and 43.3% of males reported that the first incident occurred after arrival. In Ethiopia, 32.3% of females and 65.9% of males who experienced sexual violence before age 18 reported that the first incident happened after arrival, and for physical violence the figures were 74.7% of females and 43.9% of males. These findings demonstrate that a sizable share of older respondents experienced initial incidents during displacement, supporting the inclusion of this age group in the analysis. We have included these findings in lines 112 to 118 and 121 to 124 of the manuscript file with track changes accepted.

We acknowledge that the cross-sectional design limits causal interpretation and have noted this in the limitations section. However, we do not recommend excluding the 18–24 age group because these respondents have completed the full childhood and adolescent period and can provide a complete account of exposure to violence before age 18. Excluding them would remove valuable information about polyvictimization across the full developmental window of interest.

Regarding the concern about “overemphasized” gender differences, our intention was to highlight that violence against children and youth exhibits clear gendered patterns in both stable and humanitarian settings, and these patterns are often intensified in camps and settlements. Presenting gender-specific results was deliberate because the risks, types of violence, and reporting patterns differ for girls and boys, and these differences are central to understanding the needs of displaced young people and thereby informing intervention strategies in a more nuanced way.

Finally, we emphasize the importance of these findings beyond addressing an academic gap. This study represents the first robust VACS implemented in humanitarian settings and provides invaluable information for key stakeholders, including the governments of Uganda and Ethiopia, UNHCR, and implementing partners, who are relying on these results to inform strategies for integrating service provision for refugee and host communities. These interventions, some of which we have supported, are critical for advancing inclusive programming. For these reasons, we respectfully request that the journal reconsider the recommendation to drop findings for the 18–24-year-old age group.

These comments have been addressed and/or clarified throughout the manuscript in tracked changes.

(2) Please describe in your methods section how capacity to provide consent was determined for the participants in this study. Please also state whether your ethics committee or IRB approved this consent procedure. If you did not assess capacity to consent, please briefly outline why this was not necessary in this case.

**Response:** Yes, both the IRBs and Research Ethics Committees approved the entire research protocol, including the consent forms and consenting process. As outlined in the *Ethical Considerations* section, respondents aged 18–24 and emancipated minors (aged 13–17 who had assumed adult roles) provided verbal informed consent. For minors aged 13–17, we obtained both verbal assent from the adolescent and parental/guardian consent. Individuals who could not understand or respond to the questions (e.g., due to cognitive impairment or severe hearing or speech disabilities) were excluded from the survey.

To further clarify, we have added a statement (lines 298 to 300 of the manuscript file with track changes accepted) explicitly noting that IRBs and Research Ethics Committees approved the entire research protocol, including the consent forms and consenting process.

3. Thank you for stating the following in the Funding Section of your manuscript: “This study was funded by the United Kingdom (UK) government through the Baobab Research Program Consortium (RPC).”

We note that you have provided funding information that is not currently declared in your Funding Statement. However, funding information should not appear in the Acknowledgments section or other areas of your manuscript. We will only publish funding information present in the Funding Statement section of the online submission form. Please remove any funding-related text from the manuscript and let us know how you would like to update your Funding Statement. Currently, your Funding Statement reads as follows:

“This study was funded by the United Kingdom (UK) government through the Baobab Research Program Consortium (RPC), with the Population Council Inc as the primary awardee under contract number PO8612. The funder did not play any role in the design of the study, collection, analysis and interpretation of the data, writing of the manuscript, or the decision to submit the manuscript for publication.”

Please include your amended statements within your cover letter; we will change the online submission form on your behalf.

**Response:** We have removed this section from the most recent draft and would like to maintain the above text – no amendment - upon publication. Thank you for making the change to the online submission form on our behalf.

**Reviewer One's Comments:**

The paper investigates the prevalence of childhood polyvictimisation and mental health outcomes, and associated risk/protective factors in Ethiopia and Uganda refugee settings. The paper uses datasets from the Ugandan and Ethiopian Humanitarian Violence against Children and Youth Surveys (HVACS). Using cross-tabulation with chi-square and estimation of a multivariate logistic regression model, the findings reveal that polyvictimisation was significantly associated with higher odds of mental health distress, self-harm, and suicidal ideation/attempts among females and males in both Uganda and Ethiopia refugee settings, respectively.

The paper is well-written and focuses on interesting issues that are prevalent in conflict-affected countries. My specific comments based on my review of the paper are provided below:

(i) The paper indicates a number of studies that focus on the prevalence of polyvictimisation in developing countries. The contributions of the paper to the existing literature could have strengthened to go beyond – undertaking another study in that focuses on humanitarian settings (Uganda and Ethiopia). The justifications provided for another study isn’t sufficient to warrant publishing the paper in a reputable journal like PLOS ONE.

**Response:** We appreciate the reviewer’s comment and would like to emphasize that, while VACS have been conducted in several developing countries, this study represents the first implementation of the VACS methodology in humanitarian settings. These survey findings help close a critical gap in understanding for Uganda and Ethiopia – countries that had previously completed national VACS, which typically exclude refugee populations. By including displaced populations, these surveys provide conclusive evidence to support their integration agendas and inform service provision for both refugee and host communities.

Furthermore, this work reflects the culmination of a global academic effort to adapt the VACS approach for humanitarian contexts, resulting in the development and application of the HVACS protocol. Completing these two surveys marks an important and novel step forward in advancing research and programming for displaced populations.

For clarity, we have added lines 98 to 106 of the manuscript file with track changes accepted, to further explain the rationale for conducting this study and to highlight that, although these refugee settings are in the Global South, they present unique enablers and barriers that can increase the risk of polyvictimization. This underscores the importance of exploring these issues in greater depth.

(ii) In the discussion of the data sources, there are some repetitions in the discussions of the data between line 138-145, and 161-164.

**Response:** Thank you for pointing this out. We have now corrected this.

(iii) In addition to using an additive index for polyvictimisation, the study could consider the use of Principal Component Analysis (PCA) or Multiple Correspondence Analysis (MCA) to generate an index for polyvictimisation.

**Response:** Thank you for the suggestion. We agree that methods such as PCA or MCA are valuable when the objective is to identify latent dimensions that capture shared variance across multiple items. However, in this study, our aim was to reflect the breadth of lifetime victimization across distinct forms of violence rather than to derive an underlying factor structure. The additive index aligns with our conceptual focus on cumulative exposure to different victimization types. This approach is widely used in the polyvictimization literature and has been applied in several published studies, which we sought to remain consistent with. It also fits the structure of our data, which rely on binary indicators for categories of violence.

(iv) In the description of mental health measure, the study generates a threshold for less severe mental health score if the mental health score is less than 13, and moderate to severe mental health stress for scores of 13 points or higher. How did the study arrive at this threshold? Please provide evidence to lend credence to the cut-off adopted.

**Response:** These thresholds have been validated in prior research, published, and are routinely applied in similar analyses. This reference was included in our article: Prochaska JJ, Sung H, Max W, Shi Y, Ong M. Validity study of the K6 scale as a measure of moderate mental distress based on mental health treatment need and utilization. Int J Methods Psychiatr Res. 2012 Jun 20;21(2):88–97.

(v) In the empirical analysis, in addition to using chi-square (X2), the paper could have used test of mean difference to obtain difference in outcomes between polyvictimisation, and non-polyvictimisation.

**Response:** Thank you for the suggestion. We agree that tests of mean differences can be useful for comparing outcomes across groups. In this study, our primary objective was to examine associations between polyvictimization status and categorical outcomes rather than differences in continuous measures. For this reason, chi-square tests were appropriate for the data structure, which relies on binary indicators for violence categories and other categorical variables. Additionally, the polyvictimization index was conceptualized as a count rather than a scale intended for mean comparison. This approach is consistent with practices in the polyvictimization literature and aligns with the study’s focus on prevalence and distribution rather than mean differences.

(vi) It would have been nice to present the econometric model used in the empirical analysis of the multivariate regression model. The econometric model should be presented such that the outcome (dependent variable), and the regressors are indicated.

**Response:** If required for this journal, we could present the econometric model to enhance clarity. We may add a description of the multivariate regression model, specifying the outcome (dependent variable) and the key regressors. The model takes the following general form:

$$Y_{i}=\beta_{0}+\beta_{1}\text{Polyvictimization}_{i}+\beta_{2}X_{i}+\epsilon_{i}$$

where:

- $Y_{i}$represents the outcomes of interest (severe mental distress, suicide ideation and/or attempted suicide, self-harm),
- $\text{Polyvictimization}_{i}$is the main explanatory variable indicating cumulative exposure to multiple forms of violence,
- $X_{i}$denotes the vector of covariates included in the analysis (age, sex, education, disability status, endorsement of IPVAW),
- $\epsilon_{i}$is the error term.

(vii) The empirical analysis could have gone beyond multivariate logistic regression to include a further empirical analysis such as propensity score matching (PSM) method that use matching of observable characteristics to obtain robust results compared to multivariate logistic regression.

**Response:** We thank the reviewer for their consideration of the analytical exploration of this data. We agree that propensity score matching is valuable when the goal is to approximate a treatment comparison by balancing observable characteristics between groups. However, our aim in this study was to examine associations rather than estimate causal effects. Logistic regression was appropriate for this purpose because it allowed us to adjust for a broad set of covariates and assess the relationship between polyvictimization and each outcome.

Additionally, VAC surveys, including our humanitarian VACS, collect a focused set of variables. While they capture violence exposure in detail, they typically include only a limited set of background characteristics such as age, sex, schooling, household composition, and a few socioeconomic indicators. These surveys are not designed for causal inference, which requires a wide range of pre-exposure confounders. For these reasons, logistic regression was the most suitable approach for our objectives and data structure.

**Reviewer Two's Comments:**

The authors utilize a novel and rare survey dataset derived from a representative sample of

children and young adults residing in refugee settlements in Uganda and Ethiopia. They examine

the risk factors associated with experiencing “childhood polyvictimization” and assess

“childhood polyvictimization” itself as a risk factor for mental illness. Using multivariate logistic

regression, the authors confirm the intuitive risk factors for childhood polyvictimization, as well

as the notion that childhood polyvictimization is a risk factor for mental illness. However, the

paper could benefit from a clearer motivation for the choice of such a narrow research question.

Unfortunately, without a strong theoretical/ex ante motivation for the research question, there

is a significant risk that it will be interpreted as an ex-post realignment of the research question

following data analysis. Below are some comments for your reference:

Major Comments

The introduction begins with a clearly defined term “violence against children (VAC)”; however, it immediately transitions to “polyvictimization” in the second sentence. There is a missing connection regarding “victimization,” which is not explicitly defined. While this may be a nitpicky point, I believe it is crucial to the framing of the paper, its contributions, connections to the literature, and welfare and policy concerns; it is important for comprehension and precision. It is only on the second page, in the middle of the last paragraph (line 108), that polyvictimization is finally explained. It would greatly enhance comprehension and motivation to move the discussion of the various forms of violence considered in this paper (physical, sexual, and emotional) to the first two paragraphs.

Since the paper centers on the core concept of childhood polyvictimization, it needs to be clearly defined and contrasted with monovictimization. Why is it necessary to separate exposure to violence, either directed towards the child or witnessed by the child, into different forms? Why is a child who suffers two episodes of sexual violence considered less victimized than a child who experiences one episode of sexual violence and one episode of physical violence? The previous two questions must be supported by psychological theory and robust empirical evidence, at least in the context of high-income countries (HIC), where data may be more abundant.

**Response:** We would like to clarify that polyvictimization may not always be operationalized as specifically considering physical, sexual, and emotional violence, as done in this paper. This is why we classified these types later in the introduction of the paper. We also have included that, with appropriate references, ‘polyvictimization is defined as experiencing several different forms of violence over a given period, is widespread and fundamentally different from exposure to a single form of victimization or repeated episodes of the same type’. Revisions made to the manuscript will further clarify this.

The analysis pools children (ages 13 to 17) and adults (ages 18 to 24), which is problematic

for the following reasons:

1. Children are asked about their current exposure to violence, while adults are asked about their exposure during childhood. These questions are used to calculate the polyvictimization score (0-5), which represents the sum of various types of violence to which individuals have been exposed.

**Response:** We were interested in examining experiences of childhood violence – before the age of 18 years – including sexual, physical, or emotional violence, by using questions posed to individuals aged 13 to 17 years on whether they had ‘ever experienced’ any of these forms of violence for lifetime exposure, and to those aged 18 to 24 years on their experiences of violence ‘before the age of 18’.

2. Close to 50% were not exposed to any violence; yet, those with a score of 0 or 1 are coded as “non-polyvictims,” while those with scores of 2, 3, 4, and 5 are coded as “polyvictims.” It seems odd to pool non-victims with mono-victims.

**Response:** Mono-victims and non-victims, as described by the reviewer, are not polyvictims, which is the focus of this paper. Our analysis specifically examines cumulative exposure to multiple forms of violence. We have, however, explored the relationships between any violence exposure (including among mono-victims) and adverse health outcomes in other papers.

3. The risk factors, such as food insecurity, are presumably contemporaneous. Thus, for adults, polyvictimization serves as a risk factor for food insecurity, as it has occurred in the past. Similarly, for adults, polyvictimization is more likely to be a risk factor for poor quality relationships with parents rather than the reverse. How is school enrollment coded for adults? Is it contemporaneous, or does it refer to their childhood?

**Response:** Thank you for raising this point. We agree that some risk factors, such as food insecurity, are contemporaneous. However, our analysis does not assume a causal direction between polyvictimization and these outcomes. The study is cross-sectional and aims to examine associations rather than establish temporal ordering or causality. While polyvictimization occurred before age 18, and outcomes like food insecurity were measured at the time of the survey, the design does not allow us to infer whether one preceded or influenced the other.

Regarding school enrollment, this variable was coded as contemporaneous, reflecting whether the respondent was enrolled in school at the time of the survey. For adults aged 18–24, this captures current enrollment status rather than childhood enrollment.

4. The term “disabled”—a significant risk factor—remains undefined. Is it disability

from birth? From forced displacement? Could adults become disabled after childhood?

Could disability result from victimization?

**Response:** Our paper clarifies that disability status (coded as 1= with disability and 0 otherwise), was assessed based on the Washington Group Short Set on Functioning (WG-SS) questions that determine whether individuals have difficulty performing basic activities such as seeing, hearing [which was excluded from the Uganda and Ethiopia HVACS], walking, cognition, self-care, and communication. For the purposes of our analysis, the sub-population ‘with disabilities’ included everyone with at least one domain that is coded as having ‘some difficulty’, ‘a lot of difficulty’, and/or ‘or cannot do it at all’

5. The paper includes “emotional violence” as a form of violence. It seems more appropriate

to classify this as verbal or psychological abuse and to reserve the term violence for physical assaults, including both sexual and non-sexual acts. It also seems problematic to group witnessing any instance of violence, whether occurring in the household or outside of it, with the previous forms of violence.

**Response:** We acknowledge that terminology can vary across disciplines. However, our use of the term emotional violence follows the standardized definitions adopted in the Violence Against Children Surveys (VACS) and related global frameworks, including those by WHO and UNICEF. These definitions classify emotional violence as acts that cause psychological harm, such as humiliation, threats, or verbal abuse, and recognize it as a distinct form of violence alongside physical and sexual violence. This approach is widely used in child protection research and programming.

Regarding witnessing violence, we agree that it differs from direct victimization. However, witnessing violence is consistently included in VACS and other global child protection studies because it is associated with adverse health and behavioral outcomes similar to those observed among direct victims. Including this measure allows for a more comprehensive understanding of children’s exposure to violence within their environment.

6. Attitudes towards intimate partner violence are deeply intertwined with exposure,

making the study of this factor as a risk for victimization less informative.

**Response:** We agree that attitudes toward IPV and exposure to violence are closely related. However, examining attitudes as a risk factor remains informative for several reasons. First, attitudes toward IPV are widely recognized in the literature as a marker of social norms and tolerance for violence, which can influence both the likelihood of experiencing and perpetrating violence. Second, these attitudes are not simply a reflection of prior exposure but can also shape future vulnerability and behavior within relationships. Including this variable allows us to capture an important dimension of the social context that may perpetuate cycles of violence.

7. In the ethical considerations section, the authors state that some of the children in the sample were emancipated. Thus, parental consent was required only for interviews conducted with non-emancipated children. In the context of household violence, this raises concerns regarding sample selection bias.

**Response:** Thank you for raising this concern. We acknowledge the potential for selection bias when parental consent is required for non-emancipated minors. However, the inclusion of emancipated minors is consistent with ethical guidelines and reflects the realities of humanitarian settings, where many adolescents assume adult roles due to displacement and family separation. Excluding emancipated minors would have systematically omitted a vulnerable subgroup with unique experiences of violence, which would bias the findings in a different way. Our sampling approach was household-based and designed to include all eligible adolescents regardless of emancipation status. That said, to minimize risk and protect participants, parents were not informed that the survey included questions on experiences of violence. This approach is standard in VACS and aligns with WHO ethical guidelines to prevent harm or retaliation against children.

Minor Comments

In all the tables, when there are only two mutually exclusive and exhaustive categories

for the variable being reported, it suffices to report the statistics for only one category,

such as “Yes.” Additionally, I am unclear about the value added by reporting the t-tests

between males and females across the various variables.

**Response:** Thank you for this observation. In our case, we included both “Yes” and “No” categories to examine differences between groups, for example, comparing those who have a supportive adult versus those who do not in relation to their experiences of violence. This approach provides a fuller picture of how protective factors and risk factors vary across gender and other characteristics, which is central to our analysis.

Regarding tests between males and females, these were included to highlight gender differences in key characteristics, which is central to our research question. Gendered patterns of violence and related risk factors are well-documented in the literature and were a critical focus of this study.

Since all the independent variables are binary, it might be more interpretable to run linear

regressions rather than logistic regressions. Alternately, instead of or in addition to the

odds ratios, the marginal effect at the mean or another easily interpretable estimate could

be provided.

**Response:** Thank you for the suggestion. We agree that linear regression and marginal effects can sometimes provide more interpretable estimates. However, in this study, our outcomes of interest were binary, and logistic regression is the standard approach for modeling such data. Logistic regression allows us to estimate the odds of an outcome occurring while adjusting for multiple covariates, which aligns with our research objectives.

We acknowledge that odds ratios can be less intuitive than marginal effects. To address this, we have presented results in terms of adjusted odds ratios with confidence intervals, which is consistent with established practice in the polyvictimization literature and global VAC studies. While marginal effects could be an alternative, they are typically used when the focus is on predicting probabilities rather than examining associations, which was our primary aim.

Since there is not much contextual emphasis, the analysis can be presented more concisely

by pooling samples from both countries and controlling for a country dummy variable.

**Response:** Thank you for this suggestion. We considered pooling samples from both countries and controlling for a country dummy variable. However, we opted to present country-specific analyses because the study aims to highlight contextual differences that are critical for programming and policy. Uganda and Ethiopia differ substantially in displacement history, settlement structures, and service environments, which influence patterns of violence and risk factors. Pooling the data would obscure these nuances and limit the practical relevance of the findings for stakeholders in each country. We have clarified this rationale in the revised manuscript in lines 322 to 324 of the manuscript file with track changes accepted.

Given the confounding associated with adult status, I recommend including interaction

terms between the adult dummy variable and each of the risk factors.

**Response:** Regarding interaction terms between adult status and risk factors, we appreciate the recommendation. While this approach could provide additional insights, our primary objective was to examine main effects and maintain interpretability given the sample size and complexity of the model. Introducing multiple interaction terms would substantially increase model complexity and reduce statistical power. We have, however, noted this as a potential area for future research.

Below are some suggestions to tighten the exposition:

1. A more concise and engaging title.

2. Parsimonious introduction of technical or technical-sounding terms. For example,

prevalence, risk, and protective factors are all restatements of the common term “risk

factors.”

consistent use of active voice. Minimal use of passive voice.

3. Repeating terms consistently. For example, with reference to violence, consistently

use “type” or “form” to ensure clarity in referencing the same concept.

**Response:** We have considered and addressed these suggestions throughout the updated manuscript.

The conclusion section is overly broad and non-specific. The paper does not claim any

causal effects and should ideally refrain from making policy recommendations based on

its conclusions.

**Response:** Thank you for this observation. We agree that the conclusion should avoid implying causality or making prescriptive policy recommendations, given the cross-sectional nature of the study. Our intention was to highlight the implications of the findings for programming and research rather than to assert causal effects. We have removed language that could be interpreted as causal or overly directive for policy.

**Reviewer Three's Comments:**

The exposition is generally good. It could be tightened in some places and expanded in others. The motivation and framing of the research question is area that requires attention. Some polishing and proof-reading could be beneficial, but it is secondary. Please see attached report for more detailed comments.

1. Clarify the relevance of the refugee context

The paper would benefit from a stronger articulation of why the refugee context is analytically important. What mechanisms or structural conditions make victimization dynamics different from those in host communities? Without this framing, readers might assume that children in refugee camps face similar risks as other disadvantaged children.

**Response:** Thank you for this important comment. We have addressed it by framing the discussion to emphasize the contrast between individuals living in camps and those outside, including host communities in lines 98 to 106 of the manuscript file with track changes accepted.

Where does the victimization primarily occur—within households, among peers, or through interactions with host-community members?

**Response:** Regrettably, our analyses of the locations where violence occurs indicate that victimization happens across all these settings and is largely perpetrated by those closest to the children, including family members, peers/schoolmates, and members of the wider community. We have explored these nuances in detail in other publications including more detailed reports for [Uganda](https://knowledgecommons.popcouncil.org/cgi/viewcontent.cgi?article=1015&context=hubs_humanitarian) and [Ethiopia](https://knowledgecommons.popcouncil.org/cgi/viewcontent.cgi?article=1026&context=hubs_humanitarian) and in our other peer-reviewed publications which can be found [here](https://knowledgecommons.popcouncil.org/do/search/advanced/?q=%22Baobab%3A%20Sexual%20and%20Reproductive%20Health%20and%20Rights%20in%20Refugee%20Settings%22&start=0&context=12095065&sort=date_desc&facet=).

If feasible, provide basic descriptive contrasts between the refugee sample and national statistics (e.g., share of female-headed households, poverty rates, school enrollment). Such comparisons would help contextualize the findings.

**Response:** We agree that national statistics can provide some context. However, we opted not to include direct comparisons for two reasons. First, the refugee populations in our study differ substantially from national populations in terms of displacement history, household composition, and access to services, and because refugee populations are not from the same country as the nationals of the host country, which would make such comparisons potentially misleading. Second, our primary aim was to examine patterns within refugee settings rather than contrast them with national averages, which would require additional methodological considerations to ensure comparability. That said, have added some of this context in lines 357 to 359 and 367 to 369 of the manuscript file with track changes accepted.

2. Clarify timing and setting of victimization

It is not entirely clear whether the episodes of polyvictimization occurred within the camps or prior to displacement. Because polyvictimization is defined as exposure before age 18, respondents aged 18–24 may have spent part of their childhood outside the camps.
- How long have these individuals been displaced?

**Response:** Thank you for raising this point. We addressed this in detail in response to the editor’s comments above, as it is indeed an important question. Our survey assessed the median duration of stay in the settlement for household heads (from households where participants were recruited). For those aged 18–24, the median duration was six years for both male and female participants in Uganda, and 11 years for females and 12 years for males in Ethiopia. This information provides context on how long respondents have lived in displacement settings and can be found in more detailed reports for [Uganda](https://knowledgecommons.popcouncil.org/cgi/viewcontent.cgi?article=1015&context=hubs_humanitarian) and [Ethiopia](https://knowledgecommons.popcouncil.org/cgi/viewcontent.cgi?article=1026&context=hubs_humanitarian).

Could the higher prevalence among older respondents in Ethiopia reflect pre-displacement experiences rather than conditions in the camps?

**Response**: This is an excellent observation. However, the results show that most of the first incidents occur after arriving in the settlements or camps. Among 18-24-year-olds (older respondents) who experienced sexual violence before age 18, in Uganda, 73.3% of females and 53.2% of males reported that the first incident of sexual violence in childhood happened after arriving in the settlement. For physical violence, 66.6% of females and 43.3% of males reported the first incident happening after arriving in the camp. For Ethiopia’s 18-24-year-olds (older respondents) who experienced sexual violence before age 18, 32.3% of females and 65.9% of males reported that the first incident of sexual violence in childhood happened after arriving in the camp. For physical violence, 74.7% of females and 43.9% of males reported the first incident happening after arriving in the camp. We agree that this clarification is crucial for interpreting the age pattern in the results.

3. Disaggregate the forms of victimization and explore gender-norm mechanisms
Polyvictimization is defined as exposure to two or more types of victimization. The paper could benefit from disaggregating these experiences to show which combinations most commonly overlap (e.g., emotional–physical, physical–sexual). Such descriptive insights would deepen understanding of mechanisms behind the associations. Moreover, the study surfaces a potentially important link between gender-norm attitudes (endorsement of IPVAW) and experiences of childhood victimization. The observed association between endorsing intimate-partner violence and being a polyvictim is intriguing yet counterintuitive. One might expect that individuals who normalize violence would under-report such experiences, yet the opposite pattern emerges. This tension might stem from the types of violence reported—certain forms (e.g., emotional or community-level) may be interpreted differently depending on gender-norm beliefs.

To probe this, the authors could produce a cross-tabulation between types of violence and respondent characteristics (gender, IPVAW endorsement, disability, household structure) or, more ambitiously, replicate the analysis by defining polyvictimization as exposure to specific pairs of violence types (2 × 2)—for example, emotional + physical, physical + sexual, etc.
Either approach would enrich the discussion and clarify whether the IPVAW relationship reflects differences in perception, reporting, or actual exposure. I would be very interested in seeing these results.

**Response:** Thank you for these excellent observations and suggestions. We also found this an important area to explore and have one paper examining the association between exposure to intimate partner violence against women (IPVAW) and violence victimization among children – Odwe, G., Obare, F., Muthuri, S. et al. Childhood exposure to intimate partner violence against women and its association with violence against children in refugee settlements in Uganda. Confl Health 19, 24 (2025). <https://doi.org/10.1186/s13031-025-00661-5> – with a similar paper focusing on Ethiopia now under peer review. We appreciate these suggestions for future and further analysis and will consider them in subsequent work.

4- Understanding the relationship between mental health and polyvictimization

The paper could more explicitly unpack what drives the association between mental health outcomes (e.g., self-harm, suicidal ideation or attempts) and polyvictimization. In Ethiopia, the point estimates drop by almost half once models are adjusted for gender, suggesting that

compositional or mediating factors may be at play.

I encourage the authors to consider a sequential adjustment approach—adding individual-level factors first, then family-level, and finally community-level variables—to assess which dimension accounts for the observed attenuation. This would provide deeper insight into which factors most strongly mediate or confound the link between victimization and mental health and would strengthen the causal interpretation of the associations.

**Response:** Thank you for these thoughtful suggestions. We agree that the proposed analyses are interesting and could provide additional insights. However, implementing them would require substantial restructuring of the paper, including revisiting the analytical framework and expanding the scope beyond what was originally intended. Our current approach is warranted given the study objectives, which were to examine associations rather than estimate causal effects. Introducing more complex modeling would shift the paper toward causal interpretation, which was not our aim.

That said, we fully recognize the value of these ideas and will take them on board for future work and explore these more advanced analyses.

Minor Suggestions

When reporting results, use neutral language such as “associated with higher/lower odds” instead of causal terms (“increased risk,” “reduced risk”).

**Response:** Thank you for your comment. We have rephrased terms that could imply causality.

Visualization: The results section is dense with tables. Adding graphical summaries (e.g., bar charts or coefficient plots) would help readers grasp key patterns quickly. For instance, Table 2 contains more information than is discussed in the text; parts of it could be moved to an appendix while the main text features a concise visualization.

**Response:** We are happy to convert the tables into alternative graphical representations if the editor(s) agree with this approach.
